# Complexity of consenting for medical termination of pregnancy: prospective and longitudinal study in Paris

**DOI:** 10.1186/s12910-018-0270-9

**Published:** 2018-05-02

**Authors:** Georges Abi Tayeh, Jean-Marie Jouannic, Fersan Mansour, Assaad Kesrouani, Elie Attieh

**Affiliations:** 1 Department of Gynecology and Obstetrics, Hôtel-Dieu de France University Hospital, Boulevard Alfred Naccache, Achrafieh, P.O. Box: 16-6926, Beirut, Lebanon; 2 Faculty of Medicine, Saint Joseph University, Beirut, Lebanon; 3 Department of Gynecology and Obstetrics, Armand Trousseau Hospital - AP-HP, Paris, France

**Keywords:** Consent, Decision-making, Fetal cardiac pathology, Medical termination of pregnancy, Prenatal diagnosis

## Abstract

**Background:**

We analyzed the patients’ perception of prenatal diagnosis of fetal cardiac pathology, and the reasons for choosing to continue with pregnancy despite being eligible to receive a medical termination of pregnancy. We also identified the challenges, the motives interfering in decision-making, and the consequences of the decisions on pregnancy, child and mother.

**Methods:**

This descriptive, prospective and longitudinal study was conducted in France, amongst pregnant women who wished to continue their pregnancy despite an unfavorable medical advice (incurable fetal cardiac pathologies). Socio-demographic data were collected through a questionnaire. Such questionnaire covered information assessing the parents/mother’s perception of prenatal diagnosis, and medical termination of pregnancy, their interpretation of the established diagnosis and their motives for not considering pregnancy termination.

**Results:**

72 eligible patients were analyzed over one year: mean age 33 ± 6.89 years, 47 patients had already given birth to ≥1 healthy child. Mean gestational age at the detection of fetal cardiac pathologies was 30 ± 4.37 weeks of amenorrhea. Patients decided to keep the child after 3 ± 1.25 consultations. 56 (77.78%) patients made their decision with their husbands and 16 made their decision alone. Reasons for declining the medical termination were culpability and responsibility (*n* = 36), ideologies and convictions (*n* = 24), mistrust and hope (*n* = 12). Newborns of 67 patients died with a mean survival duration of 38 days.

**Conclusions:**

Patient informed consent should be sought before any decision in neonatology, even if conflicting with the medical team’s knowledge and the pregnant mother’s benefits. Decisions to accept or decline pregnancy termination depend on the patients’ psychological character, ideologies, convictions, and mistrust in the diagnosis/prognosis, or hope in the fetus survival.

**Electronic supplementary material:**

The online version of this article (10.1186/s12910-018-0270-9) contains supplementary material, which is available to authorized users.

## Background

Management of medical treatment requires decisions based on the gravity and complexity of the medical condition. As such, no common measures are applied for patients whether they decline an immediate salvage therapy (e.g. urgent transfusion or caesarian), or a set of therapeutic options proposed by the physician in less immediate and preoccupying situations.

Declining a treatment not only involves the patient, a fetus, a family, or the entire society in case of public health issues, but also physicians, psychologists, and the clergy. This multidisciplinary approach may lead to conflicts of interests between the third-party persons and the patients. Consequently, each medical situation should be considered on a case-by-case basis. In such cases, the patients’ autonomy in taking decisions depends on the physician, medicine, society and beliefs. In parallel, this autonomy controls any decision that does not comply with the medical logic. Further, patients make their decisions after consenting, without being unfortunately able to confirm whether the consent would provide enough information to conciliate between the current medical situation and the required knowledge for decision-making.

In this context, the primary objective of the current study was to attempt to analyze the patients’ perception of the prenatal diagnosis, namely the fetal cardiac pathology. We also aimed to analyze their reasons for declining medical termination of pregnancy, to identify the challenges and motives interfering in decision-making, and to identify the consequences of the decisions on the pregnancy, child, mother, both parents, and society.

This study covers women wishing to continue their pregnancies in spite of a medical advice confirming a life-limiting fetus because of a life-limiting cardiac untreatable illness.

## Methods

### Study design and population

This was a descriptive, prospective and longitudinal study conducted at *Hôpital Necker – Enfants Malades* in Paris, France. Eligible participants were pregnant women who decided to continue their pregnancy despite an unfavorable medical advice because they were carrying fetuses with incurable cardiac pathologies (cardiac pathology included left ventricular hypertrophy, atrioventricular communication and complex congenital cardiopathy). All cases had isolated cardiac pathologies and syndrome cases were excluded. The women were initially assisted for information and advice provided unanimously by obstetricians, neonatologists and cardio-pediatricians. For the purpose of the study, they were also followed up for 4 years between April 2004 and April 2008 in the maternity ward of the hospital.

### Study site

Eligible women had different origins, social conditions, and convictions. They were referred to *Hôpital Necker – Enfants* Malades in Paris, France, which is a national reference center in cardio-pediatrics, and the only reference center to treat fetal cardiac pathology in Ile-de-France region. That ensured a better representativeness of the study population compared with other centers that recruit patients from the same area or neighborhood. It is noteworthy that the region of Ile-de-France covers a large population with 11 million inhabitants registered in 1997, which is equivalent to 236,845 pregnancies leading to 162,032 live births and 74,813 dead births, according to the French College of Fetal Echography.

### Data collection tool

Data were collected through a questionnaire by three obstetricians who were well trained to conduct a semi-structured interview. Such an interview helped in open questions evaluating the parents or the mother’s perception of the prenatal diagnosis and the medical termination of pregnancy, their interpretation of the established diagnosis, as well as their motives for not considering the pregnancy termination. Closed questions also allowed collecting information about the quantitative aspects of the parents’ decision (Additional file 1).

The semi-structured interview was adopted because it offered many single-choice questionnaires, it described the practice and the opinion of healthcare professionals involved in the prenatal diagnosis and assisting the parents in taking their decision. To assess its comprehensibility and feasibility, the study questionnaire was first tested over five people included in the study.

The evaluation of the responses was performed for certain complex cases by a committee of physicians in charge of the prenatal diagnosis, a psychologist, and occasionally a social assistant.

### Data collected

The data collected were divided into four groups. The first set of closed questions collected general and socio-demographic data about the couples, and the medical, surgical, gynecological, and obstetric history of the mother. A low socioeconomic level corresponded to an annual income of 15,000 Euros or less (approximately 18,630 USD/year), middle level to an income between 15,000 and 35,000 Euros per year (18,630 to 43,470 USD/year) and high to more than 35,000 Euros per year. The second part of questions reported information about the diagnosis, such as the conduct of the prenatal diagnosis, fetal ultrasound and echocardiography, time for birth when the fetal pathology was diagnosed, and disease prognosis and severity as reported in the medical file. They also collected any information provided before ultrasound and after the fetal pathology’s diagnosis, and information relevant to how, when and who disclosed the diagnosis, to how parents were assisted to decide whether to terminate or not the pregnancy, and to the number of consultations, as well as information disclosed and undisclosed by the patients. The third set of questions recorded data about decision-making (causes of the decision, identity of the decision-maker, and influence of a third-party person). Finally, the fourth part collected information before and during labor (patient’s support, number of consultations, consultants), as well as data relevant to the pregnancy and the newborn’s outcome (duration of hospitalization, number of consultations, consequences of the parents’ decision on their lives).

### Content analysis

The semi-structured questionnaire was first recorded after the participant gave her consent, and then entirely transcribed to the database. Content analysis was performed using a reading grid where the keywords and main ideas of each response to all questions were reiterated, and where interviewees’ responses were compared at a later stage.

### Ethical considerations

The study was approved by the ethical committee of the participating center before initiating the study. All participants were interviewed after signing a written informed consent. Data collection and analysis were performed. Patients were entitled to their autonomous decision and their anonymity was protected.

### Statistical analysis

The study was both qualitative and quantitative for more validity. The explanatory factors and the motives of the decision were analyzed qualitatively, while the pregnancy, mother, child, and parents’ characteristics were analyzed quantitatively. All data were analyzed with SPSS software for Windows Release, version 16 (SPSS Inc. Released 2007. SPSS for Windows, Version 16.0. Chicago, SPSS Inc.). Continuous variables were described using means and standard deviations, qualitative variables were expressed as numbers and percentages.

## Results

### Socio-demographical characteristics and medical history

The study recruited 72 eligible patients over one year in the maternity department of *Hôpital Necker – Enfants Malades*. The mean age of the patients was 33 ± 6.89 years, ranging from 23 to 44 years old. The social, cultural and economic levels were low in 47 (65.27%) patients, while they were middle in 16 (22.22%) patients, and high in 9 (12.51%) patients. As for the professional activity, 47 (65.27%) out of the 72 participants were active whereas 25 (34.73%) patients were housewives. Also, 28 (38.89%) patients had French origins.

None of the participants had consanguineous marriage with the father of the fetus, and 47 (65.27%) patients had already given birth to at least one healthy child.

### Diagnosis of fetal pathologies

**Fig. 1:**
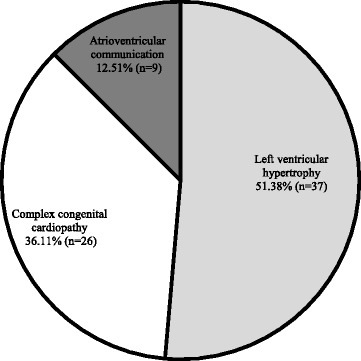
Cardiac pathologies diagnosed in the fetuses (*n* = 72)

The mean gestational age at the detection of the fetal pathology was 30 ± 4.37 weeks of amenorrhea (range: 24 to 37 weeks), where the diagnosis was incurable cardiac pathologies for all fetuses (Fig. 1).

### Decision-making

The patients made their decisions to keep the child after an average of 3 ± 1.25 consultations (range: 1 to 5 consultations), where 30% of the participants had already asked for a medical advice in another center. Also, 2 (2.78%) patients sought advice from the clergy before making their decisions. 56 (77.78%) patients made their decisions with their husbands, two of whom did not speak French. In those cases, the husbands translated for their wives during the interviews and confirmed the determination of their spouses to keep the child. We could not communicate with the patients to be sure of their will. The remaining 16 (22.22%) patients made their decision alone.

Reasons for declining the medical termination of pregnancy were classified into three groups, namely (1) culpability and responsibility, (2) ideologies and convictions, and (3) mistrust and hope. Patients of the first category (*n* = 36, 50%) did not want to interrupt their pregnancy because they mentally conceived an affective relationship with their fetuses, and could not tolerate any active and aggressive procedure leading to the feeling of guilt. They did not want to accept any responsibility with regards to the ending of the life of their child, and preferred to let nature take its due course. As for the second group, it consisted of 24 (33.33%) patients who declined a medical termination of their child’s life for religious beliefs and convictions. In the third group, 12 (16.67%) patients believed that their child could exceptionally survive after birth.

### Labor and newborn’s outcome

In total, 4 (5.56%) patients had a cesarean delivery, while 68 (94.44%) patients delivered their babies vaginally, three of whom requiring forceps delivery. Two women had vaginal tears which were repaired without complications, and 36 (50%) patients asked not to undergo any cesarean as would be indicated by the fetus’ condition.

**Fig. 2:**
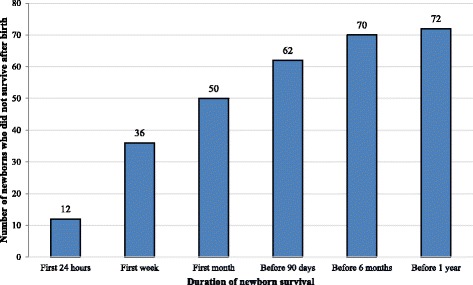
Neonatal mortality (*n* = 72)

As for the newborns, 5 (7%) patients were lost to follow up after the delivery. Nonetheless, all newborns of the 67 remaining patients died with a mean survival duration of 38 days ranging from 1 min to 1 year (Fig. 2). Also, 24 (33.33%) patients had previously declined any neonatal resuscitation while 8 (11.11%) patients declined any therapeutic obstinacy during neonatal resuscitation. Newborns were entitled to post-natal palliative care without a post-natal end-of-life decision during the newborn survival period.

## Discussion

The ethical principles of beneficence and respect for autonomy were the essence of our study. This ethical principle obligates the obstetrician-gynecologist, like all physicians, to seek the greater balance of clinical benefits over clinical harms for the patient as a consequence of the physician’s behaviors [1]. 72 patients wishing to continue their pregnancy in spite of life-limiting fetal cardiac pathologies were included in our study. 72 babies were born with a 100% mortality rate ranging from 1 min to a year, with an average of 38 days of life. The medical advice about the life-limiting conditions was confirmed after birth. The wish of the parents to continue the pregnancy was accepted in all cases, with all the consequences of this wish on the mother and her pregnancy on the medical team and on the society.

As for the parties involved in decision-making, the consent to any medical act is crucial for the patient’s care management. It is also related to the principle of respecting the integrity and dignity of the human person, as stated in the Universal Declaration of Human Rights [2], the European Commission for Human Rights [3], and the Charter of Fundamental Rights. Accordingly, health care professionals should respect the people’s rights to the intangibility of their own body. No one can harm them without their consent even for curative purposes, as indicated by the article 36 of the French Code of Medical Deontology [4]. Nevertheless, it is more difficult nowadays to get the patients’ consent given that they are less confident in medicine since they are getting their medical information from a multidisciplinary team, including medical and non-medical parties. In this sense, the dilemma between the physicians’ will and the patients’ wish has always existed. From one side, the physicians have always believed that their decisions are in the patients’ interest. Whereas from the other side, the modern medical ethics broke this paternalistic tradition where the patients can accept or decline the medical care, putting the physicians in a difficult situation with respect to their role in dispensing medical services, and to their medical knowledge [4]. However, the French law authorizes women to obtain a pregnancy termination until the end of the pregnancy. This requires the opinion of two experts confirming a severe and an incurable abnormality.

In fetal medicine and neonatology, patient information and consenting have particular features since all diagnostic and therapeutic procedures are performed on the mother; the fetus is totally passive and his parents are deciding instead. Another feature is that fetal death through pregnancy termination can be suggested as the only therapeutic option, which the patient can accept or decline. Consequently, any decision must be duly pondered and discernment well-developed in order not to regret or feel guilty about any decision taken. Also, the mother or couple’s decision about voluntary interruption of pregnancy can be influenced by many factors, such as doubt or hope, the couple’s active or passive character, secular or religious beliefs, and the influence of the society.

Basically, the physician and his/her team should support the patient and the couple who decided to decline the pregnancy termination. They should also ensure that the patient and her partner are aware of all necessary information, understand the foreseeable consequences of their decline, and are freely making their decision with no constraints. In this context, the performance of the medical team is based on their professional experience, keeping their medical knowledge up to date along with ethical considerations and human, socio-cultural and psychological dimensions of the patients [5]. In addition, the availability of decision-making tools and evidence-based medicine can assist the medical team in decision-making [6].

In case of non-curable and life-limiting fetal cardiac pathologies, the ethical paradox about medical termination of pregnancy is obvious amongst the partisans of termination of pregnancy who want to avoid an impaired quality of life of the newborn, and amongst those who believe in “all life equally being sacred” [7]. While both parties prioritize the respect of human life, the first is basically concerned with the intensity of life, while the second is more interested in its duration.

Once the decision to terminate the pregnancy is made, the medical team and the parents should not question the ethical aspects but the reasons. Will the fetus suffer from termination of pregnancy? Will it suffer more if pregnancy is not terminated? What might happen to the medical team, the parents and the newborn in case of live birth? Regardless of the termination of pregnancy requirements, it should be performed humanly during the second and third trimesters of pregnancy. While termination of pregnancy sounds a painful word, it is law compliant because it stops the fetus’ heartbeat without causing pain when done correctly. Therefore, the parents should be informed that the fetus anesthesia is mandatory so it does not suffer. This would help them feel less guilty. Morphine is mainly used for anesthesia followed by the injection of potassium chloride [8]. For the mother, the major caveat of this procedure is that potassium chloride can rarely but severely disseminate into the maternal circulation affecting her psychologically [9, 10]. Ultimately, the medical team, including the midwife, the obstetrician, the pediatrician and the psychologist, should respect the couple’s decision to decline the procedure, and accompany them in the pre- and post-natal periods [11].

### Declining medical termination of pregnancy

Our findings enabled us to classify the reasons for declining any medical termination of pregnancy into three major groups. First, the patients wanted to avoid feeling guilty and being responsible for ending the life of their fetus. Their affection and love for the child prevented them from terminating the pregnancy and saved all from inevitable pain and suffering. Guilt is related to affective ambivalence according to Carl Gustave Jung [12]. It is also perceived by Lewis Engel and Tom Ferguson as a misdirected and excessive feeling of altruism [13]. As for the medical team, they should relativize the decision of terminating the pregnancy because due to a life-limiting and non-curable cardiac pathology, as an attempt to relieve the couple from moral suffering. The team should also focus on the scientific and natural aspects of affection and help the couple to accept the facts and to be satisfied with their decision for the sake of the fetus.

Second, 33.33% of the patients did not ask for a medical termination of their child’s life for religious beliefs and convictions. Those are ideas and doctrines that were transmitted during the parents’ childhood, and that vary across societies. As such, whenever the human being cannot act according to their values and beliefs, they would feel spiritually distressed, guilty, anxious or doubtful regarding the meaning of their existence. In our study, two Muslim patients declined the termination of their pregnancy after consulting a religious authority, and the medical team had to respect and accept the patients’ will even if it was not in tune with the team’s scientific knowledge.

Third, 16.67% of the patients declined to terminate their pregnancy because they mistrusted the diagnosis and/or prognosis, or had hopes that their child could survive. Unfortunately, no survival exception was recorded in our study. Fetal cardiac pathologies are rare, have different levels of severity, and are reported in small series in the literature; hence, evidence-based medicine is impossible with such pathologies. Also, a study by Shub et al. about fetal cardiac pathologies showed that one of 89 diagnosis was erroneous [14], while the risk of prognosis error was bigger [15].

Finally, we observed that the patients declined medical termination of pregnancy at the end of a pregnancy. In our study, the mean gestational age was 30 weeks of amenorrhea. Zlotogora deliberates that the gestational age is the main determinant of the patients’ decision [16], and could be related to affection which intensifies as they get to delivery term. Also, women are more prone to decline the medical interruption when they are aged less than 23 or more than 40 years old, while they can accept it when they are aged less than 35 years old with a medical history of termination of pregnancy [17].

### Consequences of declining or accepting the medical termination of pregnancy

The medical termination of a pregnancy has consequences on the newborn, the mother, the medical team, and the society. The newborn might suffer from unreasonable medical obstinacy for neonatal resuscitation. Thus, ethical questions arise: “Should reanimation be unnecessarily pursued to prolong the newborn survival while the final outcome is inevitably known fatal?”, “Should we stop neonatal reanimation for a newborn whose tests show severe lesions that are responsible for severe disabilities?” Ideally, it would be better to avoid any obstinacy while respecting the parents’ will and their newborn dignity.

Fetal abnormalities are often diagnosed during the second trimester of pregnancy when most of the pregnancy complications can be avoided if medical termination of pregnancy is to be performed. Sometimes, declining termination of pregnancy can expose the mother to non-justified risks, and the medical team to the clash between the patient’s wish and her well-being. However, the team will have to comply with the wish of the mother as expressed in her consent.

As for the medical team and society, the major burden would be the economic cost of medical obstinacy, the stay in the reanimation unit costing 1500 euros (approximately 1860 USD). An additional tax was even suggested to be added to those who do not write a living will in which they declare not to undergo any therapeutic obstinacy. The aims are to reduce the cost of medical care, to prevent any quality of life’s impairment, and to ensure equity towards the medical team [18]. Also, the availability of beds in the reanimation ward is challenging for the medical team, as it would be unethical not to admit newborns with good prognosis diseases because beds are occupied by newborns with incurable diseases for resuscitation with no survival chances.

However, in case the patient consents and agrees to undergo a medical termination of her pregnancy, the medical team should be aware of the psychological effects of her decision, such as moral suffering and sorrow, which recede with time or with a new pregnancy. Korenromp et al. followed up 254 patients for 2 to 7 years after termination of pregnancy, 17.3% of whom suffered from post-traumatic psychological sequelae [19]. Those were more intense with an advanced gestational age in patients with low intellectual levels and in those who did not receive their partner’s support.

### Medical team role in decision-making

Given the consequences of declining or accepting the medical termination of pregnancy, the medical team should revise the patient’s consent and make sure that all information was correctly and accurately provided. The team should be highly qualified to study each case by itself and to counterbalance the consequences of the patient’s decision. For this purpose, we developed a decision-making tool focusing on four categories of questions addressed mainly for the patients to define their decision, their role in decision-making, the factors making the decision difficult to take, and to develop an action plan (Table 1).

**Table 1:** Decision-making tool

1. Define your decision.	ᅟ
a. What decision should you take? b. What drives you to take this decision? c. When should the decision be made? d. What are your reflections on this decision?	
2. Define your role (patient or couple) in decision-making.	
a. I prefer to decide after considering other peoples’ opinions. b. I prefer to take my decision with a third-party. c. I prefer the decision to be taken by someone else.	
3. Define the factors that make decision-making difficult.	
The difficulty in making your decision can be due to four factors: 1) Your knowledge of the options. 2) Your knowledge of what is important for you. 3) Your perception of the support or help you will receive from others. 4) Your level of certainty about your choices.	
The following questions can help you identify the factors that make your decision-making difficult:	
1) My knowledge of the options a. Are you well informed about your situation to make a decision? b. Do you know the positive and negative aspects of the available options? 2) What is important for me a. Do you know the advantages and disadvantages that matter the most for you? 3) The support I receive from the others a. Do you get enough support from the others to make a decision? b. Are you making your decision without any pressure from others? 4) My level of certainty a. Do you exactly know what your best choice would be?	
4. Develop an action plan	
Choose the factor(s), that you think are making your decision-making difficult: a. Not enough information on my options, their positive and negative aspects b. Not enough information on the advantages and disadvantages c. Not sure about the advantages and disadvantages that matter the most for me d. Lack of support or necessary resources to take my decision e. Pressure from the others to make one decision instead of another	

## Conclusions

To conclude, it is obvious that the diagnosis of fetal pathologies raises ethical issues, mainly that medical interruption of pregnancy is the only available therapeutic option in many cases. Whatever the solution is, it is mandatory for the medical team to get the patient informed consent. In neonatology, consenting is particular since it is not made by the concerned patient (fetus) but by its mother or parents. Any decision made in this context is irreversible and can require the possibility of life ending. Therefore, decisions tend to be divided between asking for or declining the pregnancy termination depending on the patients’ psychological character, ideologies and convictions, and their mistrust in the diagnosis/prognosis or hope in the fetus survival, in spite of the medical confirmation of the life-limiting condition. In our study, all fetuses died within one year from birth. Also, the medical team is confronted with a situation where they should respect the patients’ will even if it was not in tune with the team’s medical knowledge and the patient’s well-being.

## Additional file

Additional file 1: Study questionnaire. (DOCX 23 kb)
