# Supplementary material for: Complexity of consenting for medical termination of pregnancy: prospective and longitudinal study in Paris
Source: BMC Med Ethics. 2018 May 2;19:33. doi: 10.1186/s12910-018-0270-9 (PMC5932824; doi:10.1186/s12910-018-0270-9)
Supplement: Supplementary file 1 — Study questionnaire. (DOCX 23 kb) [file 12910_2018_270_MOESM1_ESM.docx]

**Original Questionnaire in French**

**(Entretien Semi Directif avec des Questions Principales après Consentement Oral)**

| **Complexité du Consentement dans l’Interruption Médicale de Grossesse**    Nom de la patiente............................................................... Date de l’entretien........................................  Adresse............................................................................. Nom de l’enquêteur...................................  Religion\ Pratiquante (oui/non)............................................ Nombre d’Enfants………………………  Origine.............................................................................. Statut marital ……………………………  **CARACTERISTIQUES SOCIO-DEMOGRAPHIQUES**  Sexe de la personne qui répond aux questions Homme_______ Femme________  Niveau d’éducation le plus élevé (choisir une réponse) :  Primaire ______________________ Autre tertiaire ____________________  Secondaire ______________________ Autre (Spécialités) ____________________    Universitaire ______________________ Pas d’éducation formelle __________________  **DIAGNOSTIC**   1. Pouvez-vous me dire avec vos propres mots le problème de votre bébé ?   __________________________________________________________________________________  __________________________________________________________________________________   1. S’agit-il de votre premier enfant ? Oui__ Non__   *Si Non*: _____   1. Vos autres enfants sont-ils en bonne santé ? Oui__ Non__ 2. Pourquoi avez-vous choisi de venir nous voir aujourd’hui ?   __________________________________________________________________________________  __________________________________________________________________________________   1. Quand avez-vous découvert l’anomalie de votre bébé ? Pendant quelle semaine de grossesse ?   __________________________________________________________________________________  __________________________________________________________________________________   1. Votre grossesse a-t-elle été suivie par un médecin et avec quelle fréquence de suivi ?   __________________________________________________________________________________  __________________________________________________________________________________   1. Combien de fois avez-vous vu votre médecin avant de prendre votre décision ?   __________________________________________________________________________________  __________________________________________________________________________________   1. Avez-vous compris ce qu’on vous a expliqué ?   __________________________________________________________________________________  __________________________________________________________________________________   1. Avez-vous demandé plusieurs avis médicaux ?   __________________________________________________________________________________  __________________________________________________________________________________   1. Avez-vous essayé d’autres types de traitement avant de venir ici ? Oui__ Non__   *Si oui:* De quel type de traitement d’agit-il ?  __________________________________________________________________________________  __________________________________________________________________________________   1. Avez-vous confiance dans le diagnostic et la sévérité de la pathologie quand les médecins les ont déclarés ? Oui__ Non__ 2. Pensez-vous qu’il est possible d’avoir un faux diagnostic en médecine ? Oui__ Non__ 3. Avez-vous décidé de garder ou pas le bébé ?   __________________________________________________________________________________  __________________________________________________________________________________   1. Si vous avez décidé de garder le bébé, pourquoi l’avez-vous fait ?   __________________________________________________________________________________  __________________________________________________________________________________   1. Avez-vous pris la décision seule ou bien avec votre partenaire ?   __________________________________________________________________________________  __________________________________________________________________________________   1. Une tierce personne a-t-elle interféré dans la prise de décision ? Oui__ Non__   *Si Oui :* Qui ? Une autorité religieuse ? Un parent ? etc…  __________________________________________________________________________________  __________________________________________________________________________________   1. Pensez-vous que le diagnostic qui vous a été donné est d’un haut niveau médical et le meilleur ?   __________________________________________________________________________________  __________________________________________________________________________________   1. Pouvez-vous expliquer pourquoi vous êtes satisfait ou pas ou comment seriez-vous plus satisfait ?   __________________________________________________________________________________  __________________________________________________________________________________  **SATISFACTION DES SOINS**   1. .Etes-vous satisfait des soins reçus ? Oui__ Non__ 2. La façon dont le bébé a été examinée ? Oui__ Non__ 3. Des médicaments administrés ? Oui__ Non__ 4. Comment s’est passé l’accouchement ? __________________________________________________________________________________   __________________________________________________________________________________   1. Comment était le soin post-natal?   __________________________________________________________________________________  __________________________________________________________________________________   1. Comment la pathologie a-t-elle évolué ? (âge de décès du nouveau-né) __________________________________________________________________________________   __________________________________________________________________________________   1. Si vous pouvez changer une chose concernant le soin ici pour l’améliorer, qu’elle serait-elle ?   __________________________________________________________________________________  __________________________________________________________________________________ |
| --- |

**English Translation**

**(Semi Directive Interview Following a Verbal Consent**

**With Some Leading Questions)**

| **Complexity of Consenting for Medical Termination of Pregnancy**    Name of patient............................................................... Date of interview........................................  Address............................................................................. Name of interviewer...................................  Religion\ Practicing (yes/no)............................................ Number of children………………………  Origins..................................................................... Marital status……………………………  **SOCIO-DEMOGRAPHIC CHARACTERISTICS**  Sex of respondent Male_______ Female________  Highest level of education (Check one answer):  Primary ______________________ Other: Tertiary ____________________  Secondary ______________________ Other (specialties) ____________________  University ______________________ No formal education __________________  **DIAGNOSIS**   1. Can you tell me in your own words what the problem is with your child?   __________________________________________________________________________________  __________________________________________________________________________________   1. Is it your first baby? Yes__ No__   *If No*: _____   1. Are all your other babies healthy? Yes__ No__ 2. Why did you choose to come to us today?   __________________________________________________________________________________  __________________________________________________________________________________   1. When did you discover the anomaly of your baby? Which week of your pregnancy?   __________________________________________________________________________________  __________________________________________________________________________________   1. Was your pregnancy followed up by a Doctor and what was the frequency?   __________________________________________________________________________________  __________________________________________________________________________________   1. How many times did you see your doctor before you made your decision?   __________________________________________________________________________________  __________________________________________________________________________________   1. Did you understand what they explained to you?   __________________________________________________________________________________  __________________________________________________________________________________   1. Did you ask for many medical advices?   __________________________________________________________________________________  __________________________________________________________________________________   1. Did you try any other kind of treatment before you came here? Yes__ No__   *If Yes:* What kind of treatment was that?  __________________________________________________________________________________  __________________________________________________________________________________   1. Did you trust the diagnosis and the severity of the pathology when doctors announced them to you? Yes__ No__ 2. Do you think that sometimes we can have a false diagnosis in medicine? Yes__ No__ 3. Did you decide to keep or remove the baby?   __________________________________________________________________________________  __________________________________________________________________________________   1. If you want to keep the baby, why did you decide so?   __________________________________________________________________________________  __________________________________________________________________________________   1. Did you decide alone or with your spouse?   __________________________________________________________________________________  __________________________________________________________________________________   1. Did someone interfere in your decision? Yes__ No__   *If Yes*: Who? A religious authority? A parent? etc  __________________________________________________________________________________  __________________________________________________________________________________   1. Do you believe the diagnosis given to you is of high medical level and the best one?   __________________________________________________________________________________  __________________________________________________________________________________   1. Can you explain why you were satisfied or not satisfied or in what ways might you have been more satisfied?   __________________________________________________________________________________  __________________________________________________________________________________  **SATISFACTION WITH CARE**   1. Are you satisfied with the care obtained? Yes________ No________ 2. The way the child was examined? Yes________ No________ 3. The drugs you were given? Yes________ No________ 4. How was the delivery? __________________________________________________________________________________   __________________________________________________________________________________   1. How was the post natal care?   __________________________________________________________________________________  __________________________________________________________________________________   1. What was the evolution of the pathology (age of death of the newborn)?   __________________________________________________________________________________  __________________________________________________________________________________   1. If you could change one thing about the care here to make it better, what would that be?   __________________________________________________________________________________  __________________________________________________________________________________ |
| --- |
